# Macrophage Migration Inhibitory Factor in Fetoplacental Tissues from Preeclamptic Pregnancies with or without Fetal Growth Restriction

**DOI:** 10.1155/2012/639342

**Published:** 2011-10-04

**Authors:** Simona Cardaropoli, Luana Paulesu, Roberta Romagnoli, Francesca Ietta, Daniela Marzioni, Mario Castellucci, Alessandro Rolfo, Elena Vasario, Ettore Piccoli, Tullia Todros

**Affiliations:** ^1^Department of Obstetrics and Gynecology, University of Turin, Sant'Anna Hospital, Via Ventimiglia 3, 10126 Torino, Italy; ^2^Department of Physiology, University of Siena, Via Aldo Moro 4 53100 Siena, Italy; ^3^Department of Molecular Pathology and Innovative Therapies, Marche Polytechnic University, Via Tronto 10/a 60020 Ancona, Italy

## Abstract

The proinflammatory cytokine MIF (macrophage migration inhibitory factor) is involved in physiological and pathological processes in pregnancy. MIF maternal serum levels are increased in preeclampsia (PE). We hypothesize that pregnancy tissues are the source of MIF overexpression in PE. MIF protein was studied in maternal sera, placental tissues, fetal membranes, and umbilical cord of 8 control and 20 PE pregnancies: 10 with normal fetal growth (PE-AGA) and 10 with fetal growth restriction (PE-FGR). MIF levels were significantly higher in PE-AGA membranes than in controls and PE-FGR. In PE-FGR, MIF cord concentrations were higher than in PE-AGA while MIF placental levels were lower than in controls. MIF maternal serum levels were higher in PE, compared to controls, and the difference was mainly due to PE-FGR samples. These data support MIF involvement in PE pathogenesis and suggest that different pregnancy tissues contribute to MIF production in PE with and without fetoplacental compromise.

## 1. Introduction

Preeclampsia (PE) is the most serious syndrome of human pregnancy and it is potentially life-threatening for both mother and fetus. In developed countries, where the diagnosis and management of the disease is a major aim of prenatal care, maternal mortality attributable to PE has been reduced. However, perinatal and long-term morbidity and neurological sequelae, due to fetal growth restriction (FGR) and/or preterm delivery, are still critical problems [1, 2]. Nowadays, there are no effective interventions to prevent or cure PE except for a timed and often premature delivery [3]. This is partly due to the fact that the aetiology and the pathogenesis of the disease are still poorly understood.

It is widely accepted that a generalized endothelial dysfunction and an exaggerated inflammatory response are involved in the pathogenesis of PE [1, 2]. Furthermore, it is assumed that an inadequate trophoblast invasion and remodelling of maternal spiral arteries may cause or contribute to the pathogenesis of the disease [4]. These tissue-remodelling processes are driven in part by placental cytokines.

Macrophage migration inhibitory factor (MIF) plays a pivotal role in inflammatory and immune diseases [5] and in inflammatory-like reproductive events as ovulation, menstrual cycle, and early placentation [6–8]. MIF was originally identified as a factor released by activated T-lymphocytes able to inhibit the random migration of macrophages *in vitro* [9]. Although macrophages and T-lymphocytes are the main sources of MIF, fibroblast, epithelial, and endothelial cells are also able to express and release MIF [10, 11]. MIF is also expressed in normal trophoblast [7] and membranes, and it is detectable in amniotic fluid and in maternal and fetal sera [12].

Traditionally, the major focus of MIF research has been on its role as a proinflammatory mediator. Indeed, it has been demonstrated that MIF directly or indirectly promotes the expression of a large panel of proinflammatory molecules, such as tumor necrosis factor (TNF)-*α*, interferon *γ*, interleukin- (IL-) 1*β*, IL-2, IL-6, IL-8, matrix metallo-proteinases (MMPs), nitric oxide, and products of the arachidonic acid pathway [5, 13].

We have previously reported that MIF maternal serum levels are increased in preeclamptic patients compared to normal pregnant women [14]. Based on these findings, we suggested that MIF might be involved in the pathogenesis of PE.

The aim of the present study was to verify the hypothesis that high MIF levels in preeclamptic maternal serum might derive from the fetoplacental unit. For this purpose we assessed the protein expression and localization of MIF in placental tissues, fetal membranes, and umbilical cords obtained from control and preeclamptic pregnancies.

## 2. Methods

We selected, classified, and managed pregnancies complicated by PE and controls whose placentae, fetal membranes and umbilical cords were processed and studied. The study was approved by our Piedmont Regional and Hospital Ethics Committee and informed consent was obtained from each woman.

Exclusion criteria were, multiple pregnancies, pregnancies complicated by prenatal or postnatal diagnosis of structural and/or chromosomal anomalies, and prepregnancy diseases (chronic hypertension, diabetes, etc.).

### 2.1. Study Population

#### 2.1.1. Preeclamptic Cases

Twenty consecutive pregnancies complicated by PE were included in our study. Preeclampsia was defined by appearance of hypertension (systolic blood pressure ≥140 mmHg and/or diastolic blood pressure ≥90 mmHg) accompanied by proteinuria (≥300 mg/24 h) after twenty weeks of gestational age in previously normotensive patients [15]. All patients had pathological uterine Doppler flow velocity waveforms (FVW), defined as a resistance index (RI) of >0.58 with or without the presence of bilateral notching [16].

Among these patients, two subgroups were identified based on the presence or absence of FGR: ten PE mothers delivered appropriate-for-gestational-age newborns (PE-AGA) and 10 PE pregnancies were complicated by FGR (PE-FGR).

The diagnosis of FGR was made according to the following criteria: ultrasound measurement of the fetal abdominal circumference below the 10th centile [17] or a growth velocity below the 10th percentile [18] and/or birth-weight below the 10th centile according to our birth-weight references [19], with abnormal FVW of the umbilical arteries [20]. All PE pregnancies were delivered by caesarean section.

#### 2.1.2. Control Pregnancies

Controls were 8 normotensive pregnancies with normal fetal growth and normal uterine and umbilical Doppler FVWs, delivered at term by caesarean section, because of breech presentation or previous caesarean section.

In all cases and controls, pregnancies were dated by an ultrasound scan in the first trimester of pregnancy.

#### 2.1.3. Clinical Parameters

The following data were collected for both cases and controls: maternal age at delivery, parity, smoking habits, body mass index (BMI = kg/m^2^) at the beginning of pregnancy, gestational age at birth, gestational age at onset of PE, mode of delivery, neonatal sex and weight at birth (neonatal weight was also expressed as Z-score, that is, exact number of standard deviations from the mean for gestational age, using our birth-weight references [19]), placental weight, uterine and umbilical artery Doppler ultrasound velocimetry indexes, blood pressure, urinary protein levels, and exposure to drugs (such as antihypertensives, corticosteroids, antibiotics, aspirin).

### 2.2. Tissue Samples

Immediately after delivery, normal and pathological placentae (umbilical cord and membranes included) were transported from the delivery room to the laboratory and, after preliminary gross examination, two series of tissue samples were obtained: (a) three full-thickness samples of placental tissue were randomly collected from an intermediate zone between umbilical cord insertion and periphery, two samples of fetal membranes were taken far away from both the free edge and the placental plate, two umbilical cord samples were dissected from an intermediate zone between insertion and fetus; each sample was fixed in neutral buffered 10% formaldehyde for 24 hours and embedded in paraffin for immunohistochemistry; (b) further three samples of placental tissue, two of fetal membranes and two of cord tissue were collected as above described, put into cryovials and immediately frozen in liquid nitrogen then stored at −70°C until tissue lysate for MIF concentration analysis by a specific ELISA assay.

### 2.3. Blood Samples

Before delivery, peripheral venous blood samples were collected in vacutainer tubes (Becton Dickinson) without anticoagulant, from mothers with normal and pathological pregnancies. Serum was separated by centrifugation immediately after clotting and stored at −20°C until assayed. Concentration of MIF in maternal serum samples were determined by a MIF ELISA assay.

### 2.4. Tissue Lysate

Tissue lysates from placenta, membrane and cord samples, from normal and pathological (PE-AGA and PE-FGR) pregnancies, were obtained after complete homogenization in RIPA buffer (50 mM Tris HCl, 150 mM NaCl, 1% (vol/vol) Triton X-100, 1% (wt/vol) Na deoxycholate, 0.1% (wt/vol) SDS, pH 7.5), and 3,000 g centrifugation at 4°C for 15 min. After total protein evaluation, tissue lysates were stored in aliquots at −70°C until assayed all together by MIF ELISA.

### 2.5. MIF ELISA

Concentration of MIF in tissue lysates as well as in maternal sera, from preeclamptic and control pregnancies were assayed by a colorimetric sandwich ELISA (enzyme-linked immunosorbent assay) as reported by Ietta et al. (2002) [12]. ELISA plates were coated with 100 *μ*L of antihuman MIF monoclonal antibody (2.0 *μ*g/mL) (R&D System) and incubated overnight at room temperature (RT). The plates were washed three times with Wash Buffer (10 mM PBS (pH 7.4), 0.05% (v/v) Tween 20), blocked by adding 300 *μ*L blocking solution (10 mM PBS (pH 7.4), 1% (wt/v) bovine serum albumin (BSA), 5% (wt/v) sucrose, and 0.05% (wt/v) NaN_3_), and incubated at RT for 1.5 h. After three washes, the samples and the standard, human recombinant MIF (R&D Systems), appropriately diluted in Tris-buffered saline-BSA (20 mM Tris-HCl, 150 mM NaCl (pH 7.3), 0.1% (wt/v) BSA, 0.05% (v/v) Tween 20), were added in duplicate (100 *μ*L/w) and incubated for 2 h at RT. The plates were then washed three times and 100 *μ*L of biotinylated goat antihuman MIF polyclonal antibody (200 ng/mL) (R&D Systems) was added to each well and incubated for 2 h at RT. The plates were washed again and streptavidin horseradish peroxidase (Zymed, San Francisco, Calif, USA) was added to each well and incubated for 20 min at RT. The plates were washed and 3,3′,5,5′-tetramethylbenzidine (Zymed) was added. After 30 min, the reaction was stopped by adding H_2_SO_4_ (0.1 M). The absorbance was measured at 450 nm using an ELISA SR 400 microplate reader (Sclavo, Siena, Italy). The MIF concentration was expressed as pg/mg of total tissue proteins, in tissues lysates, and as ng/mL, in serum samples. The sensitivity limit was 18 pg/mL. Intra- and interassay coefficients of variation (CV%) were 3.86 ± 0.95% and 9.14 ± 0.47%, respectively.

### 2.6. Immunohistochemistry

Paraffin sections from placenta, membrane and cord samples were analyzed for MIF expression by immunohistochemistry (IHC). From each sample, serial sections of 3 *μ*m were obtained, mounted on 0.01% poly-lysine coated glass-slides, and air-dried for 24 h at 40°C.

IHC was performed using the Strept ABComplex/AP method. Sections were dewaxed, rehydrated, and washed in Tris-buffered saline [TBS; 20 mM Tris-HCl, 150 mM NaCl (pH 7.6)]. Antigen retrieval was carried out by incubating sections in sodium citrate buffer (10 mM, pH 6.0) in a microwave oven at 750 Watts for 5 min for three times and preincubated with normal rabbit serum to prevent nonspecific bindings. Slides were incubated overnight at 4°C with an anti-human MIF monoclonal antibody (R&D System Abingdon, UK), diluted 1 : 100 in TBS. Slides were washed and incubated with rabbit anti-mouse biotinylated antibody (DAKO, Copenhagen, Denmark) at a dilution 1 : 200 for 40 min. The alkaline phosphatase reaction was revealed by Sigma Fast (Sigma Aldrich, St. Louis, Mo, USA) as substrate. Sections were contrasted with Mayer's Hematoxylin, mounted, and examined under a light microscope. For each case, a negative control was obtained by using the antibody preadsorbed with the recombinant MIF at the concentration of 20 *μ*g per mL of diluted antibody.

### 2.7. Statistical Analysis

Patient age, BMI, gestational age, neonatal and placental weight, birth weight Z-score, placental weight/neonatal weight ratio, blood pressure readings, and MIF concentrations were reported as mean and standard deviations (SDs). Means among groups were compared using a one-way analysis of variance (ANOVA). Tamhane post hoc tests, chosen to account for unequal variances, were calculated to identify significant differences between the dependent variables at *α* < 0.05. Categorical and nominal values (parity, smoking habit, urinary protein levels, exposure to pharmaceuticals) were analyzed by the chi-squared test (*χ* ^2^). Fisher's exact test was used for small sample sizes. A value of *P* ≤ 0.05 was considered significant. Statistical evaluation was performed using SPSS 18 for Windows (SPSS, Chicago, Ill, USA).

## 3. Results

### 3.1. Study Population

The three study groups, controls, PE-AGA, and PE-FGR, were comparable for maternal age, pre-pregnancy BMI and percentage of patients receiving antibiotics (Table 1).

All PE pregnancies differed from controls for gestational age at delivery, neonatal birth weight, and neonatal weight Z-score, blood pressure, urinary protein values and percentage of patients receiving corticosteroids or antihypertensives (Table 1). Smoking mothers were more frequent in PE (statistically significant in PE-FGR versus controls *P* < 0.036) than in controls and placental weight was significantly lower in PE with FGR (*P* < 0.001), while percentage of nulliparae was lower and placental weight/neonatal weight ratio was significantly higher in PE group with appropriate-for-gestational-age newborns (PE-AGA) compared to controls (*P* = 0.041 and *P* = 0.018, resp.) (Table 1).

The two subgroups of pregnancies complicated by PE did not significantly differ for percentage of nulliparae and smoking mothers, for gestational age at delivery, gestational age at onset of PE, blood pressure, and percentage of patients receiving drugs. In the PE-FGR group, neonatal and placental weight and neonatal weight Z-score were lower than in PE-AGA; moreover, there were differences between the two groups in urinary protein levels, umbilical and uterine artery Doppler ultrasound velocimetry indexes (Table 1).

### 3.2. MIF Concentration in Fetoplacental Tissues and Maternal Sera

**Figure 1:**
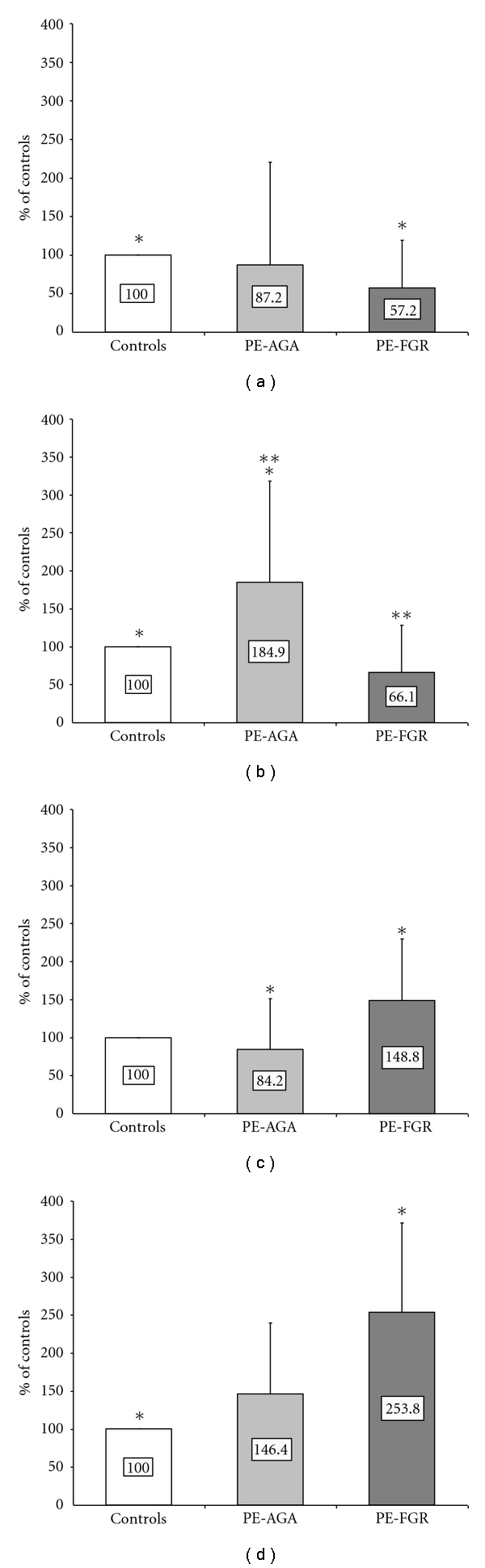
MIF ELISA assay in normal and pathological samples. (a) placenta, *P < 0.001 (control tissues versus PE-FGR); (b) fetal membranes, *P = 0.03 (control tissues versus PE-AGA) and **P = 0.004 (PE-AGA versus PE-FGR); (c) umbilical cord, *P = 0.047 (PE-AGA versus PE-FGR); (d) maternal serum,*P = 0.023 (CTRL versus PE-FGR). *P* values were calculated by ANOVA test, followed by Tamhane test for pair-wise comparison.

Quantification of MIF protein by a specific ELISA assay revealed differences between pathological and normal control samples as described herein (Table 2, Figure 1).

MIF concentration was higher in placental tissues than in fetal membranes and umbilical cords in control pregnancies (Table 2). This scenario completely changed in the two subgroups of pregnancies complicated by PE. MIF concentration was higher in fetal membranes in PE-AGA and in umbilical cord in PE-FGR as compared to the other tissues. As for PE-AGA, the difference was significant between fetal membranes versus umbilical cord (*P* = 0.005) while in PE-FGR, it was significant between umbilical cord and fetal membranes (*P* = 0.038) (Table 2). No significant differences were found in tissues when PE pregnancies were considered all together (with or without FGR) (Table 2).

Figure 1 shows comparison in MIF concentration between pathological and normal control pregnancies in fetoplacental tissues and maternal serum samples. PE values are reported as percentage of controls. MIF levels in placental tissues were significantly lower in PE pregnancies with FGR compared to controls (*P* < 0.001) (Figure 1(a)). In fetal membranes, MIF concentration was significantly higher in PE-AGA compared to normal membranes and PE-FGR samples (*P* = 0.03 and *P* = 0.004, resp.) (Figure 1(b)), while PE-FGR umbilical cord tissues had higher MIF concentration compared to levels measured in PE-AGA (*P* = 0.047) and controls (not significant) (Figure 1(c)). Finally, the increase of MIF levels in PE maternal sera was mainly due to PE-FGR samples, in fact MIF concentration in PE-FGR sera resulted significantly higher compared to control samples (*P* = 0.023) (Figure 1(d)). No significant difference was observed for any tissue when PE pregnancies were considered all together except for maternal serum: MIF maternal serum levels in PE pregnancies were significantly higher (5126 ± 2902 ng/mL) than in controls (2467 ± 703 ng/mL, *P* = 0.020).

### 3.3. MIF Immunoreactivity in Normal and Pathological Tissues

**Figure 2:**
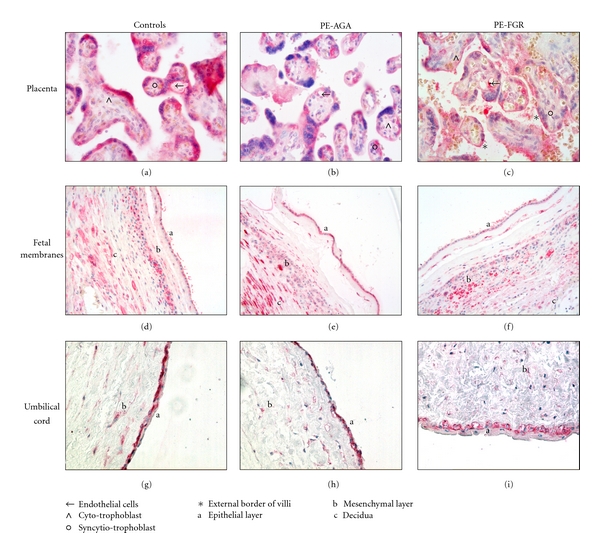
MIF immunoreactivity in tissues from normal and pathological pregnancies. Reddish color indicates positive staining for MIF. Representative images for placenta, fetal membranes, and umbilical cord from normal (a, d, g), PE-AGA (b, e, h) and PE-FGR (c, f, i) pregnancies, respectively. (←): endothelial cells; (**^*∧*^**): cytotrophoblast; (**°**): syncytiotrophoblast; (*): external border of villi; a: epithelial layer; b: mesenchymal layer; c: decidua. Original magnification: 40x for placenta panel, 20x for fetal membranes and umbilical cord panels.

Localisation of MIF by immunohistochemistry showed MIF protein in the villous trophoblast and fetal endothelial cells both in controls and PE placental tissues (Figures 2(a), 2(b), and 2(c)). Differences between control and pathological tissues were due to the appearance of a MIF immunoreactivity in PE-FGR intervillous space, mainly at the external border of villi (Figures 2(a) and 2(c)).

All cell types of fetal membranes were positive for MIF (Figures 2(d), 2(e), and 2(f)). PE-AGA fetal membranes showed a stronger MIF immunostaining of epithelial cells of amnion side and decidual cells (Figure 2(e)) compared to controls (Figure 2(d)) and PE-FGR (Figure 2(f)).

Immunohistochemistry of umbilical cord tissue sections revealed MIF expression in all cell types, and immunostaining resulted stronger in external epithelial cell layer. No distinguishable differences were observed between normal and pathological cord tissues (Figures 2(g), 2(h), and 2(i)).

## 4. Discussion

In the present study we confirmed our previous findings [14] showing that MIF maternal serum levels are higher in PE patients compared to normal pregnant women. Moreover, we investigated MIF protein concentration and localization in the placenta, fetal membranes, and umbilical cord in order to clarify the possible role of fetoplacental tissues in determining higher MIF serum levels in PE patients. Our data on MIF immunoreactivity in normal term placentas and fetal membranes confirm previously published works [12, 21], whereas our observations in the umbilical cord support *in vitro* studies conducted on human umbilical vein endothelial cells [22]. Herein we show for the first time to our knowledge that MIF is expressed in both control and PE fetoplacental tissues.

In this paper, we did not find differences in MIF concentration in tissues obtained from normal and PE pregnancies, when PE pregnancies were considered all together (with or without FGR). Nevertheless, when PE pregnancies with normal fetal growth and fetoplacental hemodynamics and PE complicated by FGR (with abnormal umbilical artery Doppler FVW) were considered separately, the picture completely changed. Placental MIF concentration was significantly lower in PE-FGR but not in PE-AGA compared to controls. MIF expression in fetal membranes was significantly higher in PE-AGA compared to both PE-FGR and controls, while MIF protein was significantly over-expressed in PE-FGR cord tissue compared to PE-AGA and higher compared to controls.

Noteworthy, the relative concentrations of MIF in placenta, fetal membranes, and umbilical cord were different among groups: they were higher in the placenta of controls, in the fetal membranes of PE-AGA, and in umbilical cord of PE-FGR. As above reported for comparison between normal and pathological pregnancies, differences were not statistically significant when PE pregnancies were considered all together. The significant difference in MIF maternal serum levels is mainly due to PE-FGR cases.

As shown in our previous study on PE, the increased serum MIF levels in PE-FGR cases were not due to *β*-methasone administration because no significant differences were detected between before and after corticosteroids treatment [14]. These findings are consistent with results by Isidori et al., (2002), showing that the response of serum cortisol to stimulation of the hypothalamo-pituitary-adrenal axis was not associated with a corresponding rise in plasma MIF [23].

It is well known that PE is a syndrome where similar symptoms could origin from different etiopathological pathways. In 2005, Redman and Sargent [1] introduced the concept of two different PE diseases: placental and maternal. Placental PE is characterized by an hypoxic placenta subjected to oxidative stress, while maternal PE arises from the interaction between a normal placenta and a maternal system susceptible or suffering of microvascular diseases, as well as long-term hypertension and/or diabetes [1]. Since FGR with abnormal umbilical Doppler FVW indicates placental compromise, we used fetal growth (FGR versus AGA) as a proxy for the definition of “placental” versus “maternal” PE. We have recently demonstrated that the enzyme HtrA1, involved in the physiological development of many organs, is differentially regulated in PE-AGA and PE-FGR placentas [24] as well as the transcription factors JunD and c-jun, implicated in regulating cytotrophoblast proliferation and differentiation, showing an opposite modulation in PE-AGA and PE-FGR [25]. Moreover, Ornaghi and colleagues observed that placental expression of anticoagulant protein Annexin 5 was significantly lower only in PE complicated by FGR—but not in PE-AGA—compared to controls [26]. Often in the literature “placental PE” and “maternal PE” are used as synonymous of “early onset” or “severe” PE and “late onset” or “mild” PE, respectively. Our study population shows that this not always true; in fact, gestational age at onset and at delivery was the same in both PE-AGA and PE-FGR groups. Moreover, severe PE complications, such as HELLP syndrome, were present in both groups.

In the light of the above observations, our findings can explain the differential role of fetoplacental compartments for increased MIF maternal serum levels in PE.

### 4.1. PE-FGR

Compared to controls, these cases were characterized by a significantly lower mean MIF protein content in the placenta. There were no difference in localization (mainly in the external layer of chorionic villi, the syncytiotrophoblast), except for the presence of MIF immunoreactivity in the intervillous space in pathological placentas.

The lower placental protein content could be the consequence of a defective translation or increased protein degradation, since MIF mRNA expression was not reduced (data not shown) among groups. An alternative explanation could be an increased MIF release in the intervillous space, thus explaining the high maternal serum levels observed in this subgroup of patients. A comparable phenomenon was observed in the epithelium of bovine epididymis and in urothelial cells of human bladder [27, 28]. In animal models of bladder inflammation and injury, it was shown that MIF protein amounts are decreased in rat urothelium and increased in the bladder lumen [28, 29]. Moreover, it was demonstrated that influenza A virus infection induces a decline of intracellular MIF protein in normal human bronchial epithelial cells, while extracellular MIF levels increases [30]. Further studies are required to demonstrate *in vitro* MIF release from PE and normal placental tissues.

The higher MIF levels that we found in PE-FGR umbilical cord were mainly localised in the epithelial and stromal cells of Wharton's jelly. FGR is due to abnormal development of the villous tree, which in turn impairs feto-maternal nutrient and gas exchanges, inducing fetal hypoxia. Since hypoxia induces MIF production [31], the low-oxygenated environment typical of PE-FGR placentas could be the most likely candidate for the increased MIF levels observed in the umbilical cord.

### 4.2. PE-AGA

This population was characterized by a significantly higher MIF expression in fetal membranes compared to controls. A similar increase of MIF immunostaining was found in fetal membranes of *Plasmodium falciparum* infected placentae [32]. Epithelial layer of membranes was previously studied as the source of MIF in amniotic fluid [12, 33] and MIF levels in amniotic fluid were increased in inflammatory conditions [34, 35]. It is plausible that fetal membranes could be the source of the slight increase in MIF maternal serum levels observed in PE-AGA.

In conclusion, our study further supports the evidence that MIF-related inflammation plays a pivotal role in the pathogenesis of PE. Our data provided new insights on the tissues responsible for the increased maternal MIF serum levels in PE, although it does not answer the question whether increased maternal MIF serum levels are the cause or the consequence of inflammation. Indeed, MIF was shown to upregulate and to be upregulated by proinflammatory stimuli [5, 36]. Of clinical relevance, we were able to discriminate between placental and maternal preeclampsia on the base of MIF source within fetoplacental tissues.

## Figures and Tables

**Table 1:** Clinical characteristics of control and pathological pregnancies.

	Controls	PE-AGA	PE-FGR	*P* value ^a^
Number of patients	8	10	10	
Maternal age at delivery (years), mean (SD)	32.8 (4.3)	34.5 (3.8)	32.5 (5.3)	n.s.
Nulliparae, number and %	7* (87.5)	5* (50)	8 (80)	*0.041
Smoking mothers, number and %	0° (0)	2 (20)	5° (50)	°0.036
Prepregnancy BMI (Kg/m^2^), mean (SD)	21.4 (2.5)	24.2 (6.1)	24.4 (4.7)	n.s.
Gestational age at delivery (weeks), mean (SD)	37.5^∗°^ (3.2)	30.6* (2.6)	30.0° (2.5)	^∗°^<0.001
Neonatal birth weight (g), mean (SD)	3254^∗°^ (439)	1360^∗∧^ (309)	1001^°∧^ (277)	^∗°^<0.001; ^∧^0.048
Neonatal weight Z-score, mean (SD)	0.26^∗°^ (0.95)	−1.02^∗∧^ (0.30)	−2.02^°∧^ (0.58)	*0.018; °<0.001; ^∧^0.004
Placental weight (g), mean (SD)	541° (81)	428^∧^ (203)	206^°∧^ (58)	°<0.001; ^∧^0.021
Placental weight/neonatal weight ratio, mean (SD)	0.17* (0.01)	0.31* (0.13)	0.23 (0.10)	*0.018
Gestational age at onset of PE (weeks), mean (SD)	n.a.	29.3 (3.1)	28.5 (3.1)	n.s.
Blood pressure (mmHg), mean (SD):				
Systolic	110.9^∗°^ (9.4)	158.4* (6.6)	150.0° (18.9)	^∗°^<0.001
Diastolic	71.8^∗°^ (7.5)	96.4* (9.5)	93.9° (9.8)	^∗°^<0.001
Proteinuria, number and %:	^∗°^	^∗∧^	^°∧^	
<1 g/24 h	0 (0)	0 (0)	5 (50)	
<5 g/24 h	0 (0)	7 (70)	3 (30)	^∗°^<0.001; ^∧^0.033
≥5 g/24 h	0 (0)	3 (30)	2 (20)	
Umbilical arterial mean pulsatility index, mean (SD)	n.a.	1.10 ^∧^ (0.17)	2.03 ^∧^ (0.32)	^∧^<0.001
Uterine arterial mean resistance index, mean (SD)	n.a.	0.67 ^∧^ (0.07)	0.76 ^∧^ (0.05)	^∧^0.021
Patients receiving, number and %:				
Corticosteroids	1^∗°^ (12.5)	9* (90)	10° (100)	*<0.001; °0.003
Antihypertensives	0^∗°^ (0)	10* (100)	10° (100)	^∗°^<0.001
Antibiotics	2 (25.0)	2 (20)	0 (0)	n.s.

PE: preeclampsia.

PE AGA: preeclamptic pregnancies with appropriate-for-gestational-age newborns.

PE-FGR: preeclamptic pregnancies with fetal growth restriction.

BMI: body mass index.

n.s.: not significant.

n.a.: not available.

^a^ *P* values were calculated by ANOVA test, followed by Tamhane test for pairwise comparison, or by chi-squared test (*χ* ^2^).

*Comparison between Controls and PE-AGA group.

°Comparison between Controls and PE-FGR group.

^∧^Comparison between PE-AGA and PE-FGR groups.

**Table 2:** Concentration of MIF in normal and pathological samples.

	Number of patients	Placental tissue (pg/mg)	Fetal membranes (pg/mg)	Umbilical cord tissue (pg/mg)	*P* value^a^
Controls	8	163,8* (112,4)	87,7 (88,1)	72,3* (46,4)	*0,034
All PE	20	119,5 (80,3)	112,8 (105,5)	85,6 (58,2)	n.s.
PE-AGA	10	142,8* (94,6)	162,2° (116,7)	60,9^∗°^ (48,4)	*0,006; °0,005
PE-FGR	10	93,7 (51,9)	58,0° (54,5)	107,5° (58,5)	°0,038

PE: preeclampsia.

PE-AGA: preeclamptic pregnancies with appropriate-for-gestational-age newborns.

PE-FGR: preeclamptic pregnancies with fetal growth restriction.

n.s.: not significant.

^a^ *P* values were calculated by ANOVA test, followed by Tamhane test for pair-wise comparison.

*Comparison between placental and umbilical cord MIF concentrations.

°Comparison between fetal membranes and umbilical cord MIF concentrations.
